# Bionic Design of High-Performance Joints: Differences in Failure Mechanisms Caused by the Different Structures of Beetle Femur–Tibial Joints

**DOI:** 10.3390/biomimetics9100605

**Published:** 2024-10-08

**Authors:** Jiandong Cui, Yubo Wang, Sen Lin, Zhiwei Tuo, Zhaohua Lin, Yunhong Liang, Luquan Ren

**Affiliations:** 1 The Key Laboratory of Bionic Engineering, Ministry of Education, Jilin University, Changchun 130025, China; cuijd22@mails.jlu.edu.cn (J.C.); wyb24@mails.jlu.edu.cn (Y.W.); liangyunhong@jlu.edu.cn (Y.L.); lqren@jlu.edu.cn (L.R.); 2 School of Mechanical and Aerospace Engineering, Jilin University, Changchun 130025, China; linsen23@mails.jlu.edu.cn (S.L.); linzhaohua@jlu.edu.cn (Z.L.); 3 State Key Laboratory of Material Processing and Die & Mould Technology, School of Materials Science and Engineering, Huazhong University of Science and Technology, Wuhan 430074, China; 4 Institute of Structured and Architected Materials, Liaoning Academy of Materials, Shenyang 110167, China

**Keywords:** beetle, failure mechanism, mechanical property, rotatable mechanism, bionic design

## Abstract

Beetle femur–tibial joints can bear large loads, and the joint structure plays a crucial role. Differences in living habits will lead to differences in femur–tibial joint structure, resulting in different mechanical properties. Here, we determined the structural characteristics of the femur–tibial joints of three species of beetles with different living habits. The tibia of Scarabaeidae *Protaetia brevitarsis* and Cetoniidae *Torynorrhina fulvopilosa* slide through cashew-shaped bumps on both sides of the femur in a guide rail consisting of a ring and a cone bump. The femur–tibial joint of Buprestidae *Chrysodema radians* is composed of a conical convex tibia and a circular concave femur. A bionic structure design was developed out based on the characteristics of the structure of the femur–tibial joints. Differences in the failure of different joint models were obtained through experiments and finite element analysis. The experimental results show that although the spherical connection model can bear low loads, it can maintain partial integrity of the structure and avoid complete failure. The cuboid connection model shows a higher load-bearing capacity, but its failure mode is irreversible deformation. As key parts of rotatable mechanisms, the bionic models have the potential for wide application in the high-load engineering field.

## 1. Introduction

With the development of science and technology, bionics, as an interdisciplinary subject, has shown great application potential in the fields of materials science, mechanical engineering, and biology [1,2,3,4,5]. Organisms in nature have evolved over millions of years to achieve a near-perfect balance between structure, function, and performance, providing rich inspiration for artificial materials and design [6,7,8,9,10,11]. With the rapid development of aerospace and other fields, high-performance rotatable mechanisms have become a research hotspot. Research on the structure and function realization mechanisms of biological high mechanical performance rotatable components can provide a new design idea for rotatable mechanisms. As an ideal bionic object of lightweight and high-strength functional materials, beetles have become a research focus on the multistage structure of their elytra and femur–tibial joints [12,13,14,15,16,17]. However, although remarkable progress has been made in the study of elytra structure in bionics research, detailed studies of beetle femur–tibial joints are still relatively scarce. The beetle femur–tibial joint is a typical representative of long-term high load, and researching it can provide a design scheme for high-performance rotatable mechanism components [18].

In recent years, with the development of bionics, beetle femur–tibial joints have been studied as natural rotational mechanisms. Some beetles have evolved digging legs because they need to dig through dirt or feces to survive. Some of the same insects have evolved clasping legs [19,20,21,22,23,24,25,26,27], grasping legs, pollen-carrying legs, swimming legs, clinging legs, and, jumping legs [22,28,29]. Differences in leg function may lead to differences in femur–tibial joint performance between beetles. The study of the femur–tibial joint structure of beetles with different living habits can illustrate the different choices of rotatable mechanisms required for varying working conditions. At present, researchers have studied the surface microstructure of leg joints of different beetles [30]. Nadein [20] systematically studied the joint self-cleaning function of *Pachnoda marginata* and showed that the anti-friction mechanism found in the leg–tibial joint is based on the lubricant spreading over the contact surface, rolling or moving under low load and deforming under high load, preventing direct contact between the joint elements [31,32,33,34,35,36]. Wear of the cuticle was detected in the beetle femur–tibial joint and its presence was experimentally confirmed under laboratory conditions [31]. The femur–tibial joint cleaning system is based on the sliding of the joint contact surface and is equipped with three structures: “hair”, “brush” and “scraper”.

We have conducted detailed studies on the femur–tibial joint of dung beetles and found that its structure is completely different from that of Scarabaeidae *Protaetia brevitarsis*, Cetoniidae *Torynorrhina fulvopilosa*, and Buprestidae *Chrysodema radians* [18]. The femur–tibial joints of other beetle femur–tibial joints with different habits can also provide different structural designs which may help to optimize bionic joints by comparing the different failure modes between different structures. We chose the femur–tibial joints from three kinds of beetles with different living habits as research objects and carried out bionic design. The different mechanical properties and failure processes undergone by these different structures can provide diversified choices for the bionic design of rotatable mechanisms.

In this study, the unique morphological and dimensional characteristics of joints of Scarabaeidae *Protaetia brevitarsis* (PB), Cetoniidae *Torynorrhina fulvopilosa* (TF), and Buprestidae *Chrysodema radians* (CR) beetles were analyzed. Specifically, the tibia of PB and TF beetles, through its cashew-shaped protrudates, achieves a smooth sliding mechanism in the guide rail composed of circular and conical protrudates on both sides of the femur. In contrast, in the design of the femur–tibial joint of CR, the two sides of the tibia are equipped with conical bumps, and the two sides of the femur are cleverly arranged with circular depressions so that the conical bumps of the tibia can be closely inserted to achieve a flexible rotation function. Inspired by these femur–tibial joint structures, we designed the bionic structures and revealed the different performances of different connection modes under failure conditions through experimental verification and finite element analysis. The experimental results show that although the spherical connection model can bear low loads, it can maintain partial integrity of the structure and avoid complete failure. The cuboid connection model shows a higher load-bearing capacity, but its failure mode is irreversible deformation. As the core components of the rotatable mechanism, the two bionic models have shown broad application potential in the engineering field requiring high loads. They not only provide novel ideas and methods for the innovative design of key components such as mechanical arms and rotatable mechanisms but also indicate important contributions in improving equipment performance and extending service life.

## 2. Materials and Methods

### 2.1. Specimen Handling and Sample Preparation

**Figure 1:**
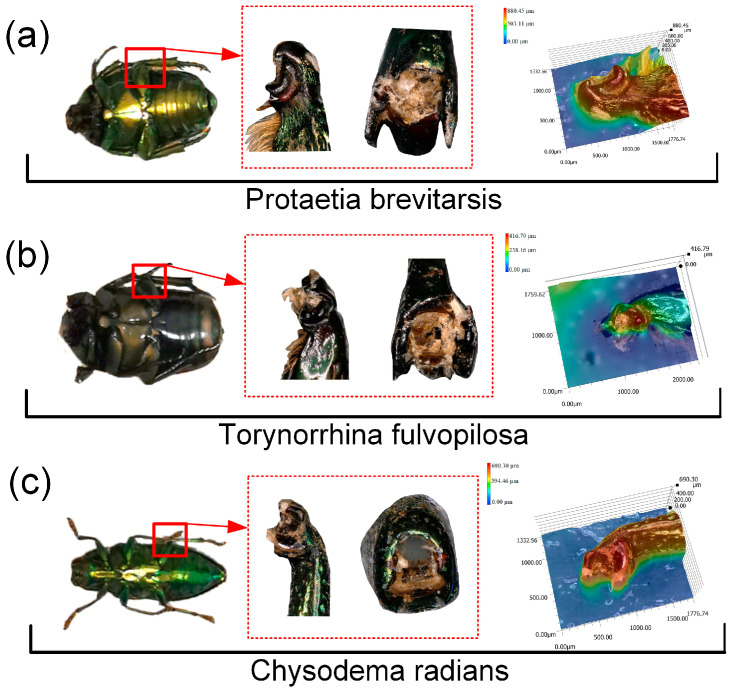
Adult, joint, and tibial terminal height cloud images of (**a**) PB, (**b**) TF, and (**c**) CR.

*Protaetia brevitarsis*, *Torynorrhina fulvopilosa*, and *Chrysodema radians* were kept in beetle boxes and fed with beetle jelly. In order to ensure the freshness of the experimental samples, the beetle femur–tibial joints were taken from euthanized adult individuals and were immediately used for experiments after the legs were removed. As shown in Figure 1, we chose the femur–tibial joint because this joint is the largest of the beetle legs. The morphology of the beetle and beetle femur–tibial joint was observed by confocal microscope.

### 2.2. Mechanical Testing

Both ends of the prepared beetle leg sample were wrapped with light curing resin, and then the light curing resin was plastically deformed into cuboid blocks on both sides to form a relatively standard dog-bone shape sample. The loading speed of the C43 MTS universal tensile tester (MTS System Corporation, Shanghai, China) in the tensile test and compression test was 5 mm min^−1^. Three samples of each beetle leg were selected for parallel testing, and the mean and standard deviation of the test results of each group of samples were calculated.

### 2.3. Micro Computed Tomography

The obtained beetle femur–tibial joint was fixed with light curing resin and placed into the device for ZEISS Xradia 610 Versa scanner Micro-CT scanning (Carl Zeiss AG, Shanghai, China). The scanning resolution of the PB femur–tibial joint was 5.0662 μm. The scanning resolution of the TF femur–tibial joint was 3.1370 μm. The scanning resolution of the CR femur–tibial joint was 3.4763 μm. Then, 3D reconstruction was performed in Mimics.

### 2.4. Digital Image Correlation Testing

Digital Image Correlation (DIC, VIC-2D) compares images before and after sample deformation to obtain deformation information of the specified region through correlation calculations. The model was prepared into a long strip stretched sample with a special sample forming mold, which was mounted on the in situ test stand for mechanical properties of biological material with a large range force sensor and used with an ARAMIS Adjustable 3D camera (Gom Metrology, Shanghai, China). The strain distribution of the photocured resin model was obtained at a loading speed of 50 μm s^−1^.

### 2.5. Finite Element

The mold was re-molded in Abaqus with 7224 and 29, 298 cells for Model 1 and Model 2, respectively. The grid property is set to C3D8R and C3D10M for Model 1 and Model 2, respectively. Due to the different structural differences between the two models, it is impossible to use a unified element size for measurement, and the excessive number of elements affects the calculation speed. Therefore, sensitivity analysis is adopted for the finite element mesh of the two models. Compared with other positions, the mesh at the contact position is dense, which makes it convenient to view the analysis result area. The minimum size of 0.3 mm is the best size, as it saves calculation time without affecting the calculation result. In order to facilitate the calculation of convergence, the rectangular edge of Model 2 is chamfered, but at the same time, the complexity of Model 2 is increased, so the C3D10M calculation is used instead of the C3D8R calculation. It was assumed that the tensile strength of the material was 55 MPa, the flexural strength was 75 MPa, and the Poisson ratio was 0.41 (data provided by the resin material supplier). Then, the bottom was set as the fixed surface of the ball head and the displacement control. The contact friction coefficient between the parts was set to 0.1.

### 2.6. Statistical Analysis

All data and error calculations were analyzed by Excel (Office 2021) and Origin (Origin 2021). Results were expressed as the mean values ± standard deviation (SD).

## 3. Results

### 3.1. Structure of Leg Joints

Figure 1a–c shows the general appearance of the beetle and its femur–tibial joint. PB feeds on fruits and young leaves and has walking legs. TF feeds on sap from tree trunks. CR feeds on trees and is fast in flight but rarely crawls, with walking legs that are thinner than the other two beetles. A special crescentic structure can be observed at the joint of the tibia and femur of the three beetles. The PB and TF femur–tibial joints are more similar to cuboid or cashew-shaped joints, while CR femur–tibial joints are more similar to conical joints. This difference in structure may lead to a large difference in mechanical properties between the femur–tibial joints of the different beetles. By selecting PB and FT, the relationship between the joint structure of the legs of similar species can be compared, and the influence of the difference of the composition materials of the legs on the results can be minimized. The ultradepth-of-field microscope allows us to take high cloud images at the ends of the tibia, providing clearer views of the structure at the joint.

**Figure 2:**
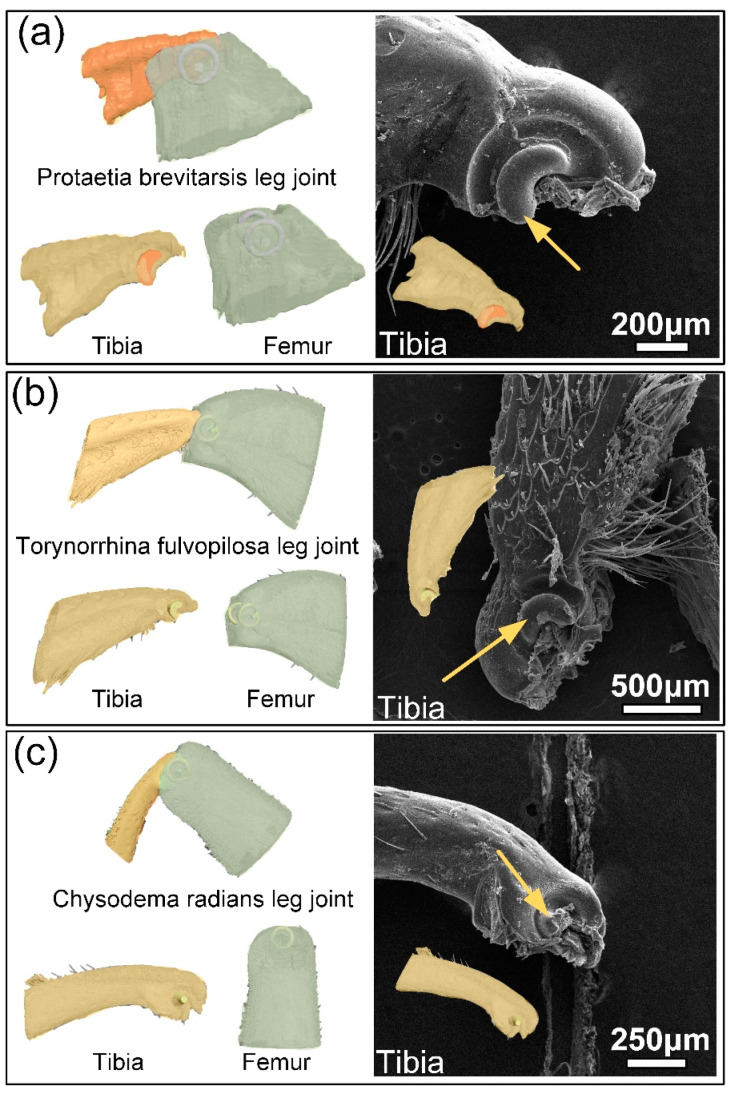
A three-dimensional reconstructive model SEM images of femur–tibial joints of (**a**) PB, (**b**) TF, and (**c**) CR.

Figure 2a–c shows the three-dimensional reconstruction models of the femur–tibial joints of three species of beetles. SEM images of the tibial end joint head with obvious structure and easy observation were taken to compare with the reconstructed models. The structural differences between the joints of the legs can be clearly seen from the SEM results. The typical structural features of the beetle femur–tibial joint were marked with yellow arrows in the SEM images. The 3D reconstruction model of the PB and TF femur–tibial joint is divided into two parts. The cashew-shaped protrusions on both sides of the tibia can be clearly seen in the SEM image, while the two sides of the femur are composed of circular and conical protrusions to form a guide rail, in which the cashew-shaped protrusions of the tibia can slide. The CR three-dimensional reconstruction model of the femur–tibial joint is divided into two parts. The conical convex on both sides of the tibia can be clearly seen in the SEM image, and there is a torus-like depression on both sides of the femur, in which the conical convex of the tibia can be inserted and rotated. The mating structure of the joint of the femur and tibia has been highlighted in different colors. PB and TF have similar femur–tibial joint structures and show little difference in mechanical properties. The structure of the CR femur–tibial joint is different from other beetles, and its mechanical properties are also poor. The difference in the structure of PB, TF, and CR femur–tibial joints may be the cause of the difference in mechanical properties. The key to revealing the differences in mechanical properties is to extract the key dimensions of femur–tibial joint through a 3D reconstruction model and construct a bionic model to verify the failure forms of the different structures. After the 3D reconstructed models of the three species of beetles were obtained, the CT models were further sliced, and the structure of the joints could be seen more clearly.

**Figure 3:**
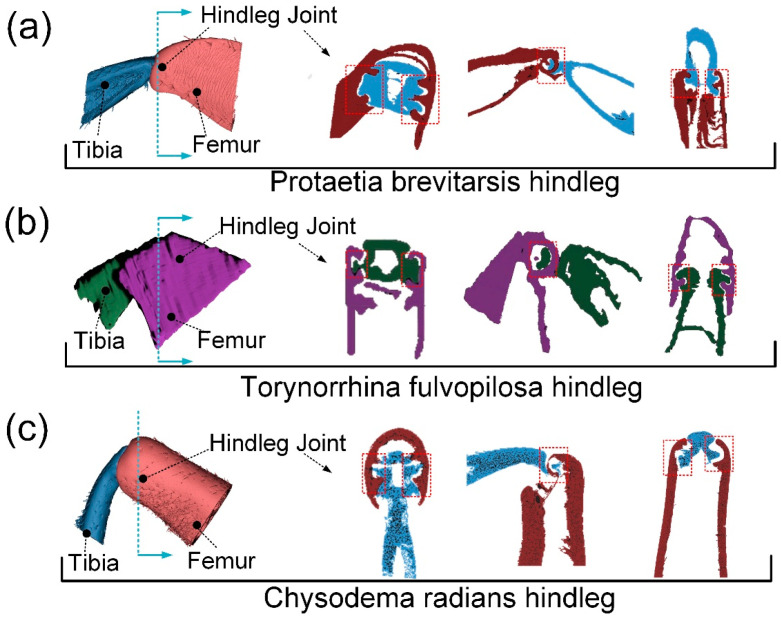
(**a**) Fish-scale microstructure on the surface of tibia segment; (**b**) brush-like microstructure on the surface of tibia segment; (**c**) laser confocal image, height cloud map, and some microstructural contour lines of the surface microstructure on the surface of femur segment with brush-like microstructure.

The femur–tibial joints of the three beetles are raised at the tibia and sunken at the femur, which is the opposite of the femur–tibial joint of dung beetles previously studied [18]. PB and TF femur–tibial joint tibial bulges are relatively long and more similar to rectangular or circular sliders. The projection of the CR femur–tibial joint is more similar to a cylindrical or conical column. The articular fossa at the end of the femur and the wrapped articular head at the end of the tibial segment can be clearly observed in the section diagram in Figure 3a–c. In the metacentric view, the femur–tibial joints of the three beetles are cylindrical or conical. In the side view, you can see the other three beetles where the femur–tibial joint joins and the tibia breaks off where the leg wraps around it, showing the presence of a slide-like structure. In the top view, the tibia protruding from the femur–tibial joint of the three beetles and the femur–tibial joint obviously form a hook-like matching structure, which is a typical cross-section structure of slide block and guide rail. Unique connections may result in femur–tibial joints with different mechanical properties. Further measurement of key parts can lay a solid foundation for the design of bionic models.

### 3.2. Mechanical Properties of Leg Joints

**Figure 4:**
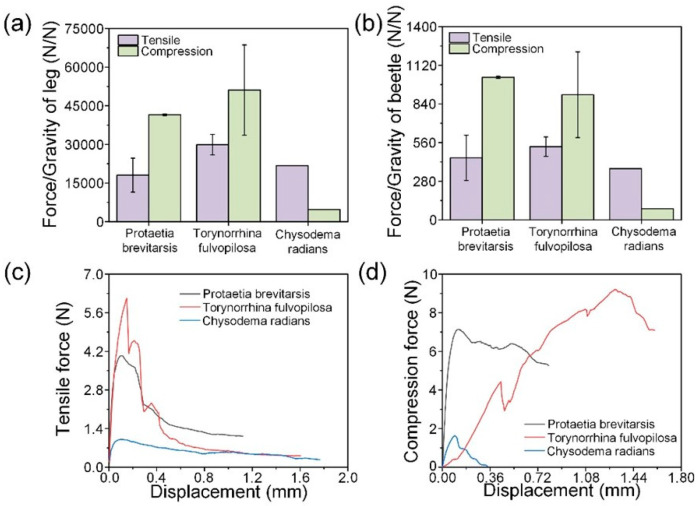
Mechanical test results of the femur–tibial joints of PB, TF, and CR. (**a**) Ratios of breaking force to leg weight for the joints of the beetles; (**b**) ratios of breaking force to beetle weight for the joints of the beetles; force-displacement curves of the (**c**) tensile tests, and (**d**) compression tests of the joints.

Figure 4a–d shows the mechanical properties of the femur–tibial joints of three beetle species. The ratio of breaking force to gravity weight was used to judge the mechanical properties in relevant papers on beetle elytra studies [13]. In the tensile test, the TF femur–tibial joint showed the best mechanical properties, and the CR femur–tibial joint had the worst mechanical properties. The ratios of tensile breaking force to leg gravity of PB, TF, and CR are 1, 8075.33 and 2, 9892.02 and 2, 1757.86, respectively. The ratios of PB, TF, and CR to the whole beetle gravity were 449.66, 530.43, and 371.36, respectively. In the compression test, the TF femur–tibial joint also showed good mechanical properties, and the PB femur–tibial joint fracture force to leg weight ratio was the largest. The ratios of compressive fracture force to leg gravity of PB, TF, and CR were 4, 1520.74 and 5, 1106.25 and 4739.34, respectively. The ratios of the compressive fracture force of PB, TF, and CR to the whole beetle gravity were 1032.91, 906.87, and 80.89, respectively. In the same force-displacement curve, it can be seen that the fracture curves of TF and PB femur–tibial joints are similar, while the fracture curves of CR femur–tibial joints are different. The force of PB and TF femur–tibial joints increases almost linearly at the initial stage of stretching, and then decreases after failure. At this time, cracks appear on the femur–tibial joints. Continue to stretch, and the force slowly decreases after the femur–tibial joint fails. CR showed a nonlinear rise in the early stage of stretching, and the force did not decrease significantly but slowly after reaching the peak. It is possible that the femur–tibial joint was directly pulled out instead of cracking. PB femur–tibial joint during compression and stretching is similar. TF femur–tibial joint force rose slowly during compression and then decreased after failure. The CR femur–tibial joint in compression first slowly rises and then slowly falls. Similar to the stretching process, the femur–tibial joint may not crack but could suffer direct damage. In order to further study the mechanism of the mechanical properties of the femur–tibial joints, the structure of the femur–tibial joints of three kinds of beetles was studied.

### 3.3. Bionic Model Design of Leg Joints

**Figure 5:**
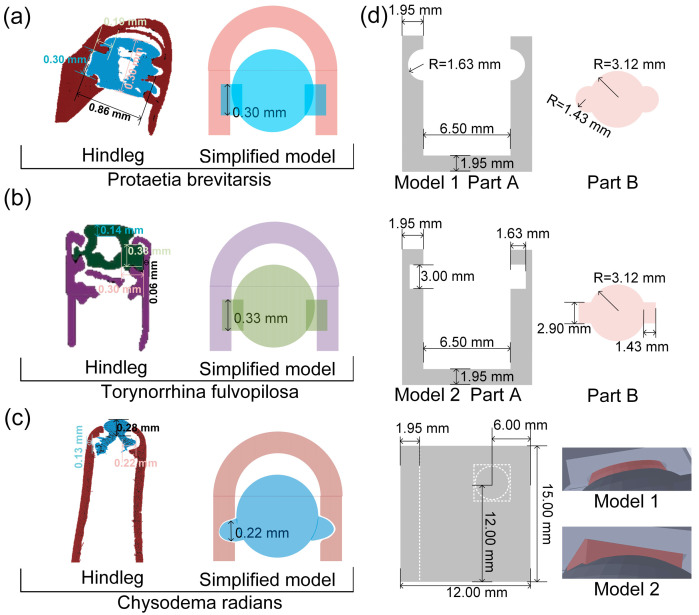
Femur–tibial joints of (**a**) PB, (**b**) TF, and (**c**) CR Micro-CT scans, and (**d**) simplified model.

In order to obtain more accurate geometric size and characteristic parameters of the beetle femur–tibial joint, the Micro-CT model obtained in the previous paper was measured. The main parameters were the wall thickness of the femur exoskeleton, the size of the femur–tibial joint, the size of the tibial joint head, the wall thickness of the tibial exoskeleton, the size of the tibial joint, etc. Figure 5a–c shows the geometric parameters of the femur–tibial joints of the three beetles. The main differences are reflected in the joint of the femur and tibia, so the main characteristic parameters are the geometric size of the joint and the size of the joint head of the tibia. The section of the PB femur–tibial joint is rectangular in shape with a length of 0.19 mm and a width of 0.30 mm. The section of the TF femur–tibial joint is rectangular in shape with a length of 0.30 mm and a width of 0.33 mm. The cross-section of the CR femur–tibial joint is a quasi-hemispherical protrusion with a length of 0.22 mm. The connection between the tibia and femur of the PB and TF femur–tibial joint is simplified as Model 1 with a cuboid connection, and the connection between the tibia and femur of the CR femur–tibial joint is simplified as Model 2 with a sphere connection. The volume control of the connection is the same, and the specific size is shown in Figure 5d. The model consists of a femur segment (Part A) and a tibial segment (Part B). In Part A of Model 1, the thickness of the side wall is 1.95 mm, the distance between the two sides is 6.50 mm, the thickness of the bottom is 1.95 mm, and the radius of the half-sphere depression at the connection is 1.63 mm. In Part B of Model 1, the radius of the main sphere is 3.12 mm and the radius of the sphere at the connection is 1.43 mm. The hemispherical protrusions and depressions of Part A and Part B cooperate with each other to form a whole. The side wall thickness of Part A of Model 2 is 1.95 mm, and the distance between the two sides is 6.50 mm. The bottom thickness is 1.95 mm, the length of the joint rectangle is 1.63 mm, and the width is 3.00 mm. In Part B of Model 2, the radius of the main sphere is 3.12 mm, the length of the joint rectangle is 1.43 mm, and the width is 2.90 mm. The cuboid bulges of Part A and Part B and the hemispherical depressions fit together to form a whole. The thickness of the back wall of Part A of Model 1 and Model 2 is 1.95 mm, and the overall height is 15.00 mm, while the distance from the center of the connection to the bottom is 12.00 mm and the distance from the front is 6.00 mm. During the mechanical test, Part B was pulled out and the model failed as a whole.

### 3.4. Failure Process of the Bionic Models

**Figure 6:**
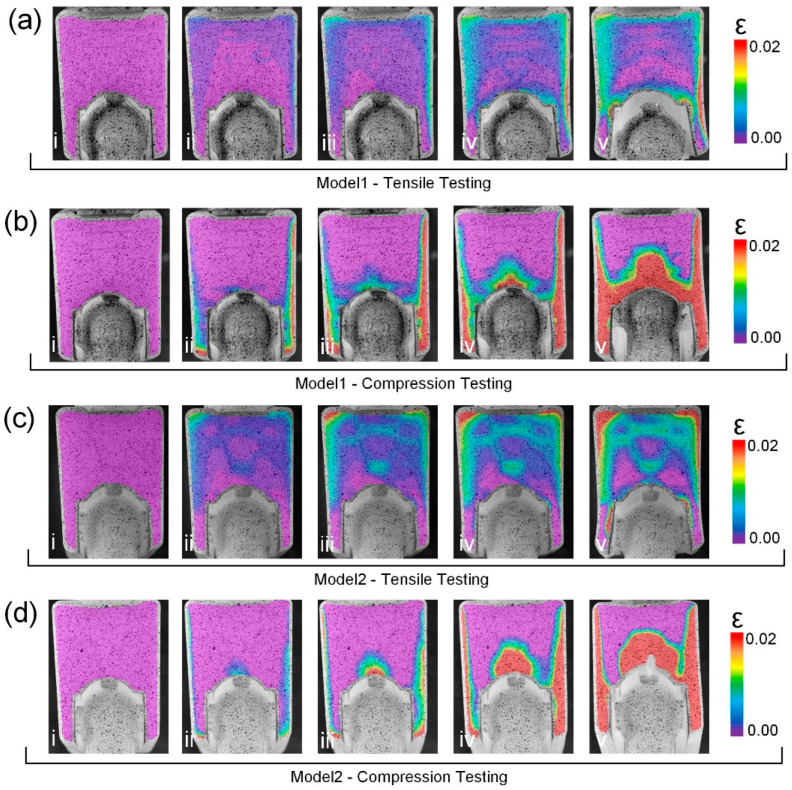
(**a**) Model 1 and (**c**) Model 2 tensile DIC process diagram; compression DIC process diagrams for (**b**) Model 1 and (**d**) Model 2.

In order to understand the influence of different structures on the failure process, the strain changes of Model 1 and Model 2 failure were studied by DIC test. In Figure 6a,b, it can be seen that when Model 1 fails in tensile and compression tests, the side wall is extruded outward to produce huge strain, and the strain is concentrated in the side wall. As can be seen from the tensile DIC test in Figure 6a, strain first occurs at the bottom of Model 1 at the initial stage of stretching. The lateral wall strain is larger in the middle stage of the tensile process. The strain is greatest at the junction between the side wall and the bottom when the final failure occurs, which may be caused by squeezing the side wall when Part B is pulled out. In the compression DIC test of Figure 6b, it can be seen that the side wall of Model 1 has a large strain in the early stage of compression. In the middle of the compression process, the lateral wall strain diffuses to the bottom. At the end of failure, both the side wall and bottom exhibited the maximum strain when Part B was compressed inward, creating a greater inward squeeze on the side walls. In Figure 6c,d, it can be seen that when Model 2 fails in tensile and compression tests, Part B is also extruded outwards to produce huge strains, which are also concentrated in the side walls. As can be seen from the tensile DIC test in Figure 6c, the bottom of Model 2 also first generated strain at the initial stage of stretching. The bottom strain is larger in the middle of the drawing process. At the end of failure, the strain at the bottom joint is the largest, and the side wall has a small strain. In the compression DIC test in Figure 6d, it can be seen that the side wall of Model 2 has a large strain in the early stage of compression. In the middle of the tensile process, the lateral wall strain diffuses to the bottom. At the final failure, both the side wall and the bottom showed maximum strain, which was similar to the last compression stage of Model 1. It can be judged that the side wall will be extruded outward during failure, and it can be seen that the changes in the tensile test should focus on the connection between the side wall and the bottom, indicating that the sample is more likely to fail from the connection between the side wall and the bottom in the tensile test. In the compression test, the strain is concentrated in the lateral wall, indicating that the lateral wall is more damaged when the joint head is removed. DIC test can only observe the strain distribution on the surface. In order to more intuitively and stereo-dimensionally analyze the difference between the two models when they fail, the stress and strain distribution of the two models during the failure process was further analyzed by finite element simulation.

**Figure 7:**
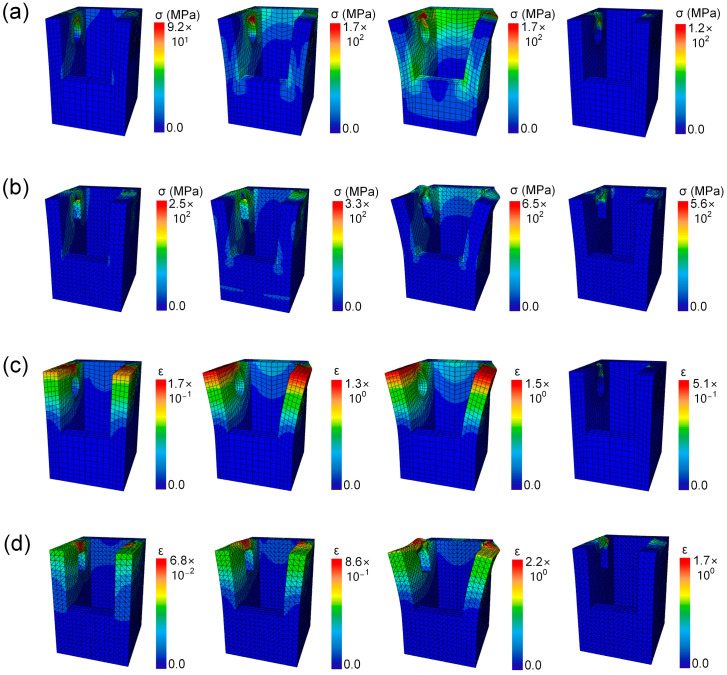
Finite element simulation stress cloud maps of (**a**) Model 1, and (**b**) Model 2. Finite element simulation strain cloud maps of (**c**) Model 1, and (**d**) Model 2.

In order to analyze the failure mechanism and the stress and strain distribution of the two models more precisely, the tensile failure process of the models was simulated by the finite element method. In order to see the stress and strain distribution of the femur segment (Part A) more clearly, the tibial segment (Part B) was hidden from the finite element simulation results. As shown in Figure 7a,b, the maximum strain of both Model 1 and Model 2 appears on the side wall, and it can be seen that the maximum stress at Part A is the part where Part B is squeezed when it is pulled out. In Figure 7a, it can be seen that the maximum stress at the initial tensile stage of Model 1 is in the hollow of the hemisphere at the joint. The side wall bends with the stretching process, and the whole side wall is subjected to greater stress. The failure of the sample produced irreversible damage to the joint. As can be seen in Figure 7b, the maximum stress at the initial tensile stage of Model 2 also appears in the cuboid depression at the joint. As the stretch progresses, the side walls bend and the joints deform outwards. The failure of the sample has caused irreversible damage to the joints and sidewalls. The damage of Model 2 is more obvious, and the side wall has produced irreversible deformation after failure. At the same time, it can be seen from the strain cloud diagram of Figure 7c,d that the strain distribution of Model 1 is almost consistent with the DIC result, but the result of Model 2 is different. Figure 7c shows that the maximum strain at the initial tensile stage of Model 1 appears at the top of the side wall rather than in the hemispherical depression. The strain distribution remains basically unchanged with the stretching. Irreversible deformation occurred at the joint after the sample failed. Figure 7d shows that the maximum strain of Model 2 at the initial tensile stage appears at the top of the side wall rather than in the hemispherical depression. The strain distribution remained basically unchanged with the stretching, but the joint protruded outwardly, and irreversible deformation occurred after the failure of the sample. The maximum strain of Model 2 is generated in the joint depression of the side wall, and DIC cannot display the three-dimensional strain, so it only shows the strain of the side wall. It can be seen that differences in the joints of the two models lead to different stress and strain distributions. The sphere connection in Model 1 is easier to pull out without completely failing the side wall. The cuboid connection of Model 2 is stronger and can withstand greater load, but it will produce irreversible deformation after failure. In engineering applications, different joint connections can be adopted according to the actual working conditions to achieve efficient design.

## 4. Conclusions

In this study, the mechanical properties and structures of the femur–tibial joints of three beetles with different living habits were studied, and bionic models were designed. DIC tests and finite element simulation of the bionic model reveal the difference in the failure process of the two models, and successfully provide different structural design options for the joints under different working conditions. The two sides of the tibia of PB and TF femur–tibial joint feature cashew-shaped protrusions, and the two sides of the femur–tibial joint are composed of a ring and conical protrusions to form a guide rail, in which the cashew-shaped protrusions of the tibia can slide. The CR femur–tibial joint tibia on both sides of the conical convex, the femur on both sides of the annular depression, and the conical convex of the tibia can be inserted in the rotation. The bionic model is divided into two types: sphere and cuboid connections. Sphere connections can withstand low loads but will not be seriously damaged after failure, while cuboid connections can withstand higher loads but will produce irrecoverable deformation after failure. This research has a wide application prospect in engineering fields such as the key components of high-load rotatable mechanisms, and provides new design ideas for engineering vehicles such as excavators and satellite solar panels.

## Data Availability

The original contributions presented in the study are included in the article; further inquiries can be directed to the corresponding author.
